# Advances in imaging-based diagnosis, prognosis, and response assessment in cardiac amyloidosis: a comprehensive multimodality review

**DOI:** 10.1007/s12149-025-02092-x

**Published:** 2025-08-25

**Authors:** Osamu Manabe, Seitaro Oda, Takashi Norikane, Tadao Aikawa, Yuka Otaki, Nagara Tamaki

**Affiliations:** 1 Department of Radiology, Jichi Medical University Saitama Medical Center, 1-847 Amanuma-Cho, Omiya-ku, Saitama, 330-8503 Japan; 2 Department of Diagnostic Radiology, Faculty of Life Sciences, Kumamoto University, Kumamoto, Japan; 3 Departments of Radiology, Faculty of Medicine, Kagawa University, Kagawa, Japan; 4 Department of Cardiovascular Biology and Medicine, Juntendo University Graduate School of Medicine, Tokyo, Japan; 5 Department of Radiology, Sakakibara Heart Institute, Tokyo, Japan; 6 Kyoto University of Medical Science, Sonobe, Japan

**Keywords:** Cardiac amyloidosis, AL amyloidosis, ATTR amyloidosis, Echocardiography, Cardiac MRI, SPECT/CT, Amyloid PET, Scintigraphy

## Abstract

Cardiac amyloidosis, characterized by extracellular deposition of amyloid fibrils within the myocardium, is an increasingly recognized cause of heart failure. With the advent of disease-modifying therapies, imaging has become central to diagnosis, subtype differentiation, prognostication, and treatment monitoring. This review provides a comprehensive update on multimodality imaging in cardiac amyloidosis, emphasizing its clinical utility across the disease continuum. Echocardiography, technetium-labeled bone scintigraphy, amyloid-specific positron emission tomography, cardiac magnetic resonance, and cardiac computed tomography each contribute uniquely to detecting amyloid burden and assessing cardiac function. In addition to outlining a practical diagnostic approach, we highlight emerging imaging biomarkers for monitoring treatment response and predicting clinical outcomes. The integration of these modalities into clinical practice enhances diagnostic accuracy, enables individualized risk stratification, and supports optimized, evidence-based care for patients with cardiac amyloidosis.

## Introduction

Cardiac amyloidosis is a progressive and often fatal condition characterized by the deposition of amyloid fibrils in the extracellular space of the myocardium, leading to restrictive cardiomyopathy and heart failure. The condition most commonly arises from either immunoglobulin light chain (AL) amyloidosis or transthyretin (ATTR) amyloidosis, the latter of which includes hereditary (variant) and wild-type forms. Early and accurate diagnosis is crucial for managing cardiac amyloidosis because treatment strategies and prognostic outcomes vary significantly depending on the type and extent of amyloid deposition [1, 2].

Imaging modalities have become indispensable in the diagnosis, risk stratification, and treatment monitoring of cardiac amyloidosis [3, 4]. Advancements in imaging techniques have markedly improved the detection of amyloid infiltration, functional assessment, and therapeutic decision-making. Echocardiography, nuclear scintigraphy with single-photon emission computed tomography (SPECT) using bone-avid tracers, amyloid-specific positron emission tomography (PET), and cardiac magnetic resonance imaging (CMR) have emerged as key tools in the non-invasive evaluation of cardiac amyloidosis [5]. In addition, cardiac computed tomography (CCT) serves as a valuable alternative for patients who are ineligible for CMR and may facilitate opportunistic screening within clinical workflows [6].

This review summarizes the latest advancements in the imaging-based cardiac amyloidosis diagnosis, focusing on key developments of the past few years. Our goal is to provide a comprehensive update on how emerging technologies are shaping the future of cardiac amyloidosis diagnostics and potentially improving patient care.

## Pathophysiology

Amyloid refers to an abnormal protein that arises when normally soluble proteins misfold and aggregate into insoluble, fibrillar structures. These fibrils deposit in tissues and organs, where they exhibit characteristic staining properties and distinctive appearances under microscopy. Because amyloid proteins are resistant to degradation, they accumulate progressively, leading to disruption of normal tissue architecture and eventual organ dysfunction [7]. The term “amyloid” was first introduced by Rudolf Virchow in the mid-nineteenth century. During autopsies, he observed abnormal deposits in organs such as the liver and spleen that reacted with iodine similarly to starch. Based on this reaction, he mistakenly believed the material to be carbohydrate in nature and coined the term “amyloid,” derived from the Latin word amylum, meaning starch [8].

Amyloidosis is a disorder characterized by the abnormal accumulation of amyloid proteins in tissues and organs. Systemic amyloidosis affects multiple organs, including the heart, kidneys, liver, and peripheral/autonomic nervous systems, often resulting in progressive dysfunction and poor outcomes. Among more than 30 identified amyloidogenic proteins, immunoglobulin light chains (AL amyloidosis) and transthyretin (ATTR amyloidosis) are the most clinically relevant in cardiac involvement. Cardiac amyloidosis is an infiltrative cardiomyopathy caused by amyloid fibril deposition in the myocardial interstitium, leading to increased wall thickness, impaired compliance, and both systolic and diastolic dysfunction [9].

In AL amyloidosis, a clonal population of plasma cells produces misfolded immunoglobulin light chains, which deposit in tissues including the heart. In ATTR amyloidosis, misfolded transthyretin (TTR), a hepatic transport protein, forms amyloid fibrils that accumulate in the heart. This condition includes two subtypes: hereditary (ATTRv), caused by TTR gene mutations, and wild-type (ATTRwt), which occurs sporadically in elderly individuals [10, 11].

## Clinical presentation

The clinical presentation of cardiac amyloidosis is often insidious and non-specific, which can contribute to diagnostic delays. Common symptoms include progressive dyspnea, fatigue, peripheral edema, and orthostatic hypotension, reflecting restrictive cardiomyopathy and evolving heart failure. On physical examination, signs of right-sided heart failure are frequently noted, including jugular venous distention, hepatomegaly, ascites, and lower extremity edema. Several extracardiac manifestations may precede cardiac symptoms and aid in early detection. In AL amyloidosis, extracardiac features such as periorbital purpura and macroglossia can serve as diagnostic clues [12]. Carpal tunnel syndrome is a well-recognized red flag for wild-type transthyretin cardiomyopathy (ATTRwt-CM), often appearing 10–15 years before cardiac involvement. Studies indicate that 30–50% of patients with ATTRwt-CM have a history of bilateral carpal tunnel syndrome, often requiring surgical release. Other extracardiac red flags include lumbar spinal stenosis and spontaneous biceps tendon rupture, both of which may result from soft tissue amyloid deposition [13]. In addition, ATTRwt-CM is increasingly recognized in elderly patients with aortic stenosis (AS), particularly those undergoing transcatheter aortic valve replacement, with a reported prevalence of 12–16% [14, 15]. These patients often present with concentric left ventricular (LV) hypertrophy and preserved LV ejection fraction (LVEF), clinical features that may overlap with AS alone.

## Treatment strategy

The management of cardiac amyloidosis depends primarily on the underlying amyloid subtype, AL-CM and ATTR-CM, and the severity of cardiac involvement. Timely and accurate typing is essential, as the prognosis and therapeutic approach differ markedly between subtypes.

In AL amyloidosis, the goal of treatment is to suppress the production of amyloidogenic light chains by targeting the underlying clonal plasma cell disorder. The current standard of care is a daratumumab-based regimen combining daratumumab, bortezomib, cyclophosphamide, and dexamethasone, which has shown significant hematologic and cardiac benefits in the phase III ANDROMEDA trial [16]. Selected patients with preserved performance status and limited organ involvement may benefit from autologous stem cell transplantation (ASCT), which can offer durable remission. Treatment response is assessed by hematologic markers such as serum free light chains and immunofixation, and by cardiac biomarkers including N-terminal pro-B-type natriuretic peptide (NT-proBNP) and troponins [17].

Therapeutic strategies for ATTR-CM have advanced dramatically in the last decade. Tafamidis, a TTR stabilizer, was the first disease-modifying therapy shown to reduce mortality and cardiovascular hospitalizations in both ATTRwt and ATTRv patients [18]. Acoramidis, another TTR stabilizer, has also demonstrated clinical benefit [19]. For patients with more advanced or rapidly progressive disease, gene-silencing therapies such as patisiran (a small interfering RNA agent) and inotersen (an antisense oligonucleotide) suppress hepatic TTR production and have shown efficacy in patients with ATTR amyloidosis with polyneuropathy, and there is growing evidence of cardiac benefits as well [18]. Most recently, in vivo CRISPR/Cas9-based gene editing has shown promising early-phase results, achieving sustained TTR suppression with a single intravenous dose [20].

For selected patients with end-stage cardiac amyloidosis and preserved extracardiac function, heart transplantation may be considered. In AL amyloidosis, transplantation is typically followed by ASCT or chemotherapy to prevent disease recurrence. In ATTRv, liver transplantation was historically used to remove the source of mutant TTR, but its role has declined with the advent of TTR silencers and stabilizers [21]. A multidisciplinary approach is essential to assess transplant candidacy and to optimize timing in relation to systemic disease control [22].

## Assessment tools for cardiac amyloidosis

Accurate diagnosis and precise subtype classification are critical for appropriate therapeutic decision-making and prognostic evaluation in cardiac amyloidosis. This section reviews the principal diagnostic modalities, such as blood biomarkers, electrocardiography, and multimodal imaging, which together form the foundation of clinical assessment.

### Blood biomarkers

Blood-based biomarkers are essential in the diagnostic evaluation of cardiac amyloidosis, providing early clues to myocardial involvement and aiding in the differentiation between subtypes. The most widely used biomarkers are natriuretic peptides, particularly BNP and NT-proBNP. These markers are released in response to myocardial wall stress and elevated intracardiac pressures, conditions that are common in cardiac amyloidosis due to restrictive physiology. NT-proBNP levels are generally higher in AL-CM than in ATTR-CM, reflecting the toxic effects of circulating light chains [23]. Cardiac troponins, particularly troponin T and troponin I, are also frequently elevated in cardiac amyloidosis due to direct myocardial injury from amyloid infiltration. In AL amyloidosis, elevated troponin levels are associated with worse prognosis and are incorporated into staging systems such as the Mayo Clinic staging algorithm [24].

In suspected AL amyloidosis, the serum free light chain (FLC) assay is indispensable. It quantifies kappa (*κ*) and lambda (*λ*) light chains, and the κ/λ ratio aids in detecting clonal plasma cell disorders. When combined with serum and urine immunofixation electrophoresis, these tests allow for the identification of monoclonal proteins, providing critical support for both diagnosis and response assessment [25, 26].

### Electrocardiography

Electrocardiography (ECG) is a key tool in evaluating cardiac amyloidosis. Low-QRS voltage in limb leads, despite increased ventricular wall thickness on imaging, is a hallmark feature due to electrical insulation by amyloid fibrils [27–29]. Pseudoinfarct patterns, such as Q waves in the absence of coronary artery disease, are also characteristic and typically indicate subendocardial amyloid deposition [2]. Conduction abnormalities, including atrioventricular (AV) block and bundle branch blocks, are common and may require pacemaker implantation. Atrial fibrillation (AF) frequently occurs and can further reduce cardiac output in the context of restrictive physiology [30, 31]. Other nonspecific findings, such as ST-segment depression and T-wave inversion, may also suggest myocardial involvement [30].

### Echocardiography

Echocardiography typically shows concentric LV thickening due to extracellular amyloid deposition rather than true hypertrophy. The myocardium may exhibit a granular or sparkling appearance on two-dimensional imaging [32, 33]. Right ventricular (RV) involvement may also be seen, with wall thickening, chamber dilation, and impaired systolic function [34].

Doppler imaging demonstrates a restrictive filling pattern, with an elevated early diastolic transmitral inflow velocity (E-wave), a diminished atrial contraction wave (A-wave), and an elevated E/A ratio. Tissue Doppler imaging further reveals markedly reduced mitral annular velocities, especially early diastolic (e') velocity, reflecting impaired compliance [35].

Speckle-tracking echocardiography allows for sensitive detection of subclinical myocardial dysfunction. A characteristic longitudinal strain pattern with preserved apical strain and reduced basal and mid-segmental strain, referred to as apical sparing, is highly specific for ATTR-CM [36]. However, its sensitivity in AL-CM is limited. A recent meta-analysis reported only moderate diagnostic accuracy of apical sparing in patients with AL-CM [37, 38].

### Nuclear scintigraphy with bone-avid tracers

Scintigraphy using bone-avid radiotracers such as technetium-99m-labeled pyrophosphate (^99m^Tc-PYP), hydroxymethylene diphosphonate (^99m^Tc-HMDP), and 3,3-diphosphono-1,2-propanodicarboxylic acid (^99m^Tc-DPD) become a cornerstone in the non-invasive diagnosis of ATTR-CM. These radiotracers have shown a high affinity for ATTR amyloid deposits in the myocardium, enabling effective imaging of cardiac involvement. The mechanism by which these radiotracers bind to amyloid deposits is not fully understood, but they are believed to have a high affinity for calcium-rich microcalcifications within amyloid fibrils [39]. Recent findings have shown an inverse correlation between the heart-to-contralateral lung (H/CL) ratio and the age of onset in patients with ATTRwt-CM, indicating different pathophysiological mechanisms depending on patient age [40].

**Fig. 1:**
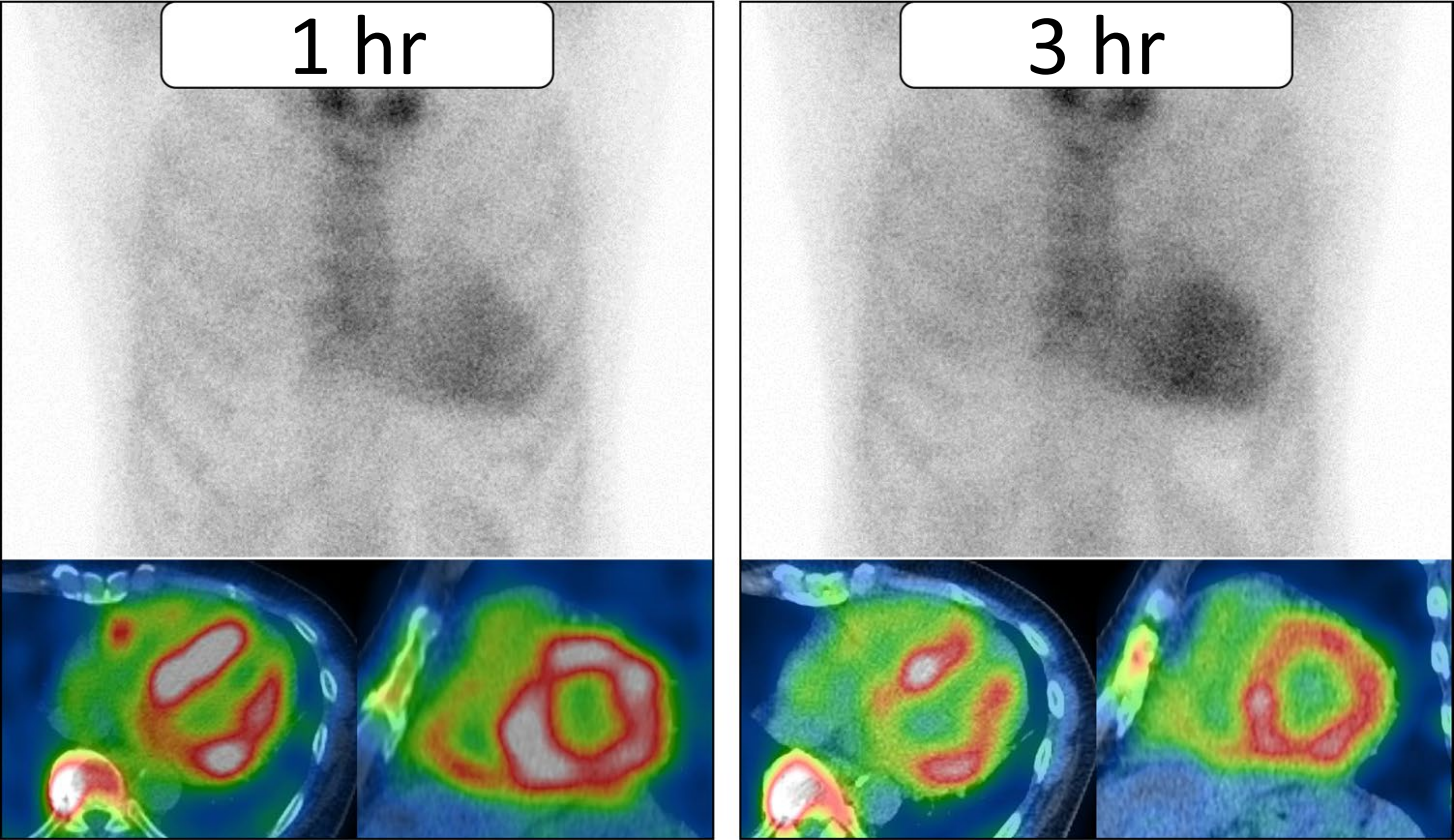
Representative case of transthyretin cardiac amyloidosis in a man in his 70 s. Planar images acquired at 1 and 3 h post-injection of ^99m^Tc-pyrophosphate (PYP), along with corresponding axial and short-axis SPECT/CT images, are shown. There is intense radiotracer uptake in the left ventricular myocardium, with relatively reduced uptake in the apical region. Mild uptake is also observed in the right ventricle on SPECT images. Visual assessment corresponds to Grade 3 (uptake greater than rib uptake with mild rib uptake). Quantitative evaluation yielded a heart-to-contralateral lung ratio of 1.88 at 1 h and 1.78 at 3 h

^99m^Tc-PYP is widely used in the United States, whereas ^99m^Tc-HMDP and ^99m^Tc-DPD are more common in Europe. In Japan, both ^99m^Tc-PYP and ^99mT^c-HMDP are used clinically**.** When interpreted using the Perugini visual grading scale (Table 1), Grade 2 or 3 myocardial uptake—especially in the absence of a monoclonal protein—is highly specific for ATTR-CM (Fig. 1) [41]. These imaging techniques are particularly valuable for distinguishing ATTR-CM from AL-CM, which typically demonstrates little to no radiotracer uptake. In hereditary ATTR-CM, ^99m^Tc-PYP scintigraphy also demonstrates moderate-to-high uptake [42]. Multiple studies and meta-analyses have demonstrated the high diagnostic accuracy of bone scintigraphy for detecting ATTR-CM [43, 44].

**Table 1:** Perugini score for bone-avid radiotracer cardiac scintigraphy

Score	Cardiac uptake (relative to bone)	Imaging findings
0	No myocardial uptake	Normal bone uptake; no visible myocardial tracer activity
1	Myocardial uptake < bone uptake	Faint myocardial uptake; ribs clearly more intense
2	Myocardial uptake = bone uptake	Myocardial uptake comparable to bone (e.g., ribs/sternum)
3	Myocardial uptake > bone uptake	Myocardial uptake clearly exceeds bone signal; ribs may appear faint

The Perugini score is a visual grading system used to assess myocardial tracer uptake relative to adjacent bone (typically ribs or vertebrae) on planar images obtained 3 h post-injection. It is widely applied across various technetium-labeled tracers, including ^99m^Tc-PYP, ^99m^Tc-DPD, and ^99m^Tc-HMDP. Grades 2 and 3, in the absence of a monoclonal gammopathy, are considered diagnostic of ATTR-CM, potentially eliminating the need for biopsy. Grade 1 is inconclusive and requires further evaluation with blood and urine tests to rule out AL-CM

A multicenter study evaluated the diagnostic performance of three technetium-labeled tracers for ATTR-CM and found that all three agents showed high accuracy [41]. When grade 2 or 3 myocardial uptake was present, the sensitivities were 94% for ^99m^Tc-DPD, 84% for ^99m^Tc-PYP, and 80% for ^99m^Tc-HMDP, while specificities were 89%, 92%, and 100%, respectively. Despite slight numerical differences, the overall diagnostic performance was comparable across tracers, particularly when combined with an absence of a monoclonal protein, yielding a specificity and positive predictive value of 100% in all groups.

Interpretation pitfalls include false-positive uptake caused by residual blood pool activity, as well as uptake in adjacent structures such as ribs, cardiac valves, or areas of prior infarction. In particular, blood pool activity can result in misleading findings on early [1, 45]. Recent data have shown that 3-h imaging significantly improves diagnostic accuracy over 1-h imaging. Recent studies have demonstrated that 3-h imaging provides significantly greater diagnostic precision compared to 1-h protocols. Furthermore, SPECT or SPECT/CT imaging should be employed to localize myocardial tracer uptake and differentiate it from surrounding structures [46–48]. While semi-quantitative indices such as the H/CL ratio have traditionally supported interpretation, their reliability is limited by factors like region-of-interest placement, overlying bone structures, and residual blood pool activity. These limitations have led to recent guidelines emphasizing visual interpretation with SPECT and SPECT/CT, especially in patients with Perugini Grade 1–2 findings.

Although AL-CM typically shows little tracer uptake, moderate-to-high uptake (e.g., Grade 2 or 3) has been observed in some cases, particularly those with coexisting myocardial injury. These findings underscore the importance of comprehensive screening for M-protein using serum and urine immunofixation and FLC assays [49, 50].

**Fig. 2:**
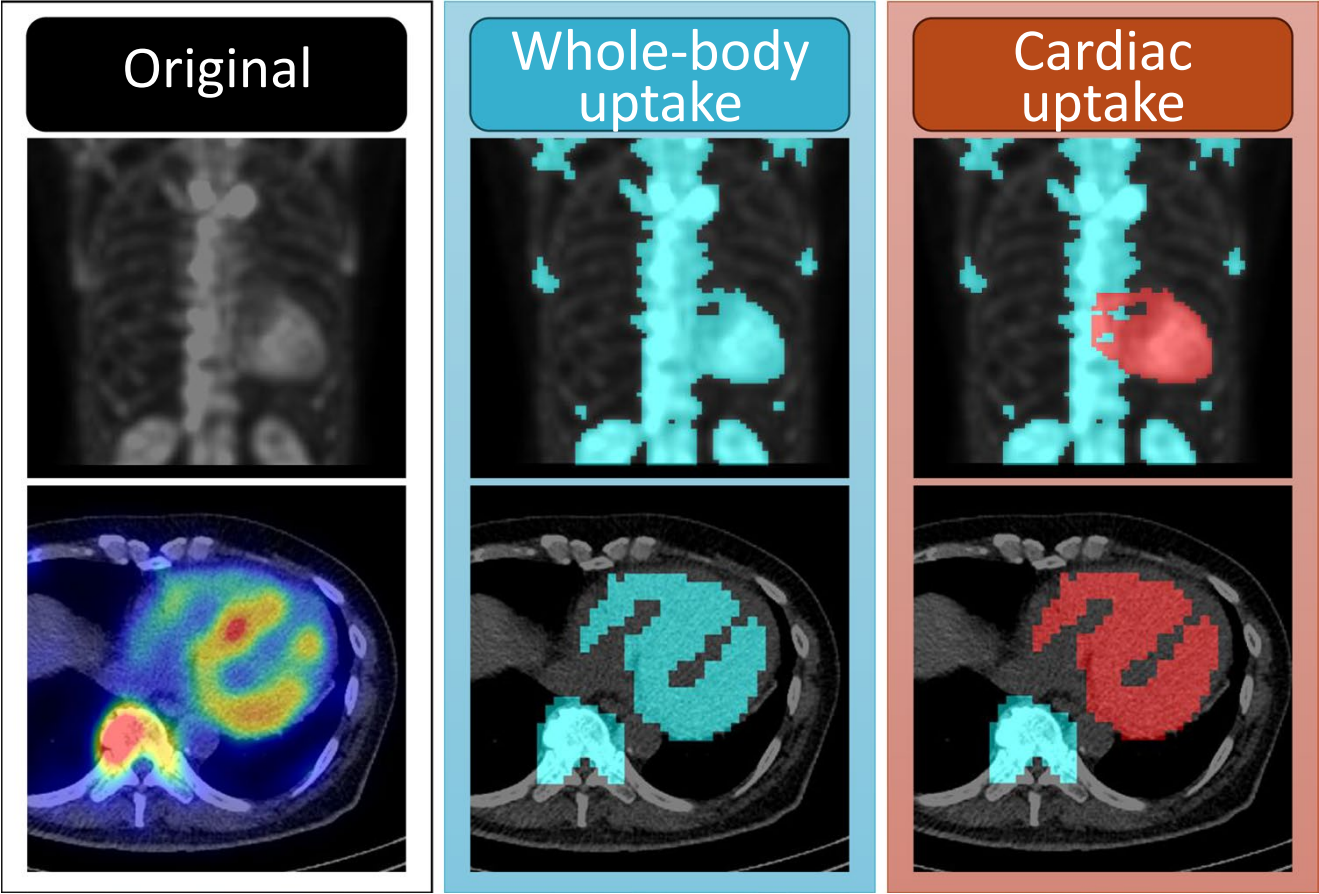
Quantitative ^99m^Tc-PYP scintigraphy in in the same patient shown in Fig. 1. Intense myocardial radiotracer uptake was observed at 3 h post-injection. The top row shows maximum intensity projection (MIP) images: the left panel displays the original image; the middle highlights all voxels above 40% of the cardiac 40% of the SUVmax (in light blue coloration); and the right isolates cardiac-specific uptake from the high-uptake regions (in red coloration). The bottom row presents corresponding axial SPECT/CT fusion images in the same order. Quantitative analysis yielded the following values: SUVmax = 6.79; amyloid deposition volume = 416.4 mL; and total amyloid uptake = 1509.3 mL

Quantitative approaches using SPECT/CT are emerging as powerful tools for measuring amyloid burden. Metrics such as standardized uptake values (SUV), total tracer volume, and percentage of injected dose can support diagnosis and track treatment response [51]. New indices, including amyloid deposition volume (AmyDV) and total amyloid uptake (AmyDV × SUVmean), show high diagnostic accuracy in hybrid SPECT/CT imaging (Fig. 2) [52]. A simple index, the lateral wall-to-aorta (LW/Ao) ratio, recently demonstrated excellent performance (area under the curve [AUC] 0.99, sensitivity 100%, specificity 97.6%) and superior reproducibility compared to the H/CL ratio and Perugini scoring [53].

Artificial intelligence (AI) applications in nuclear imaging offer further advances. AI-driven segmentation and SUV extraction have improved reproducibility and reduced observer variability. These tools correlate well with Perugini scores and cardiac biomarkers, offering promise for response monitoring and risk stratification [54, 55].

### Amyloid-specific PET tracers

**Fig. 3:**
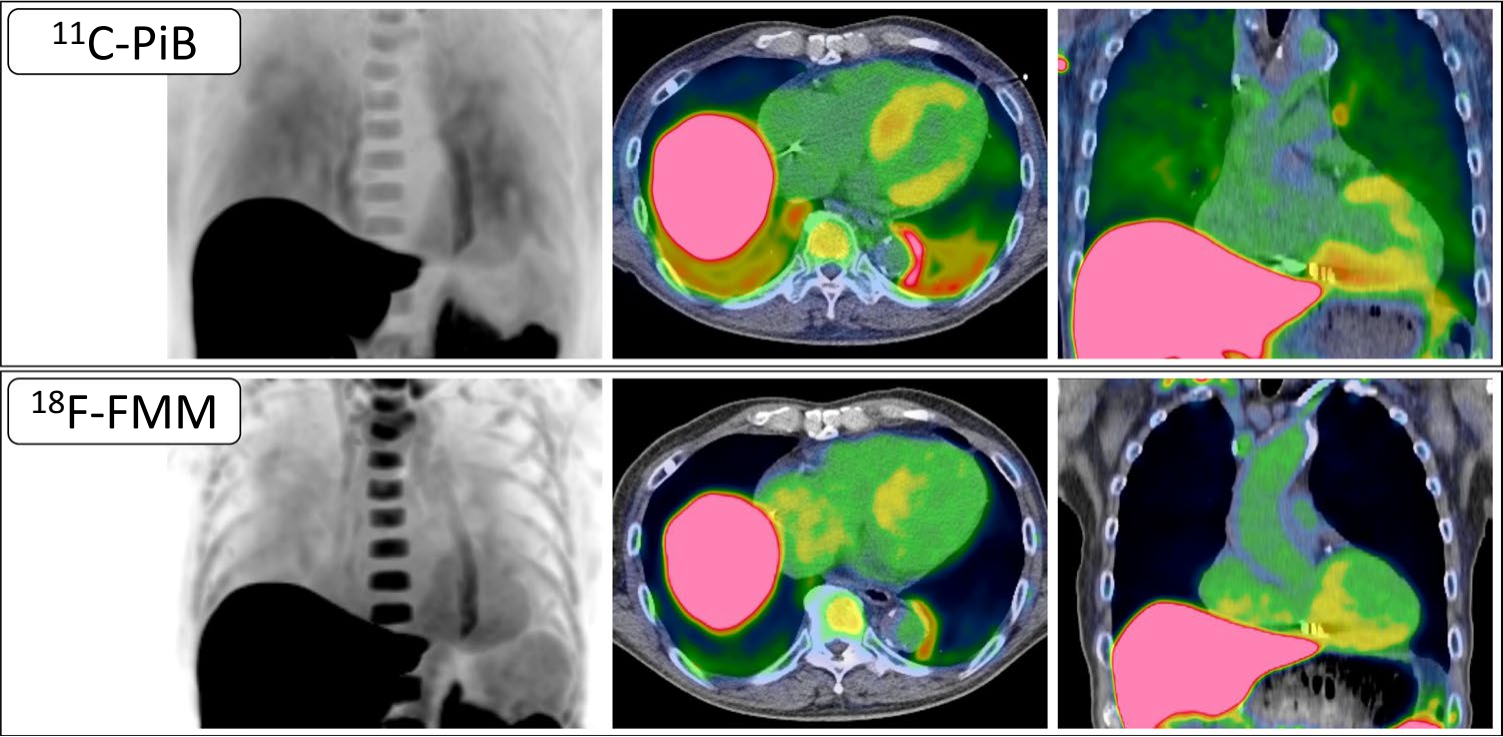
Representative PET imaging of transthyretin cardiac amyloidosis. Representative amyloid PET images from a male patient in his 80 s with wild-type transthyretin cardiac amyloidosis. The upper row shows images with ^11^C-Pittsburgh compound B (PiB), and the lower row shows images with ^18^F-florbetaben (FMM). From left to right: maximum intensity projection, axial PET/CT, and coronal PET/CT images. Both tracers demonstrate myocardial uptake predominantly in the basal segments of the left ventricular wall

Amyloid PET is an emerging imaging modality that enables non-invasive visualization and quantification of myocardial amyloid deposits (Fig. 3). It uses radiotracers that bind specifically to amyloid fibrils. The first successful Aβ-selective PET tracer, ^11^C-Pittsburgh compound B (PiB), is a derivative of thioflavin, an amyloid-binding fluorescent dye initially developed for imaging cerebral amyloid in Alzheimer’s disease. Its discovery led to the development of ^1^⁸F-labeled tracers such as florbetaben, florbetapir, and flutemetamol, which have longer half-lives and are more suitable for broader clinical use [56, 57]. Although currently investigational in cardiac amyloidosis, growing evidence supports the diagnostic utility of amyloid PET [58, 59].

**Fig. 4:**
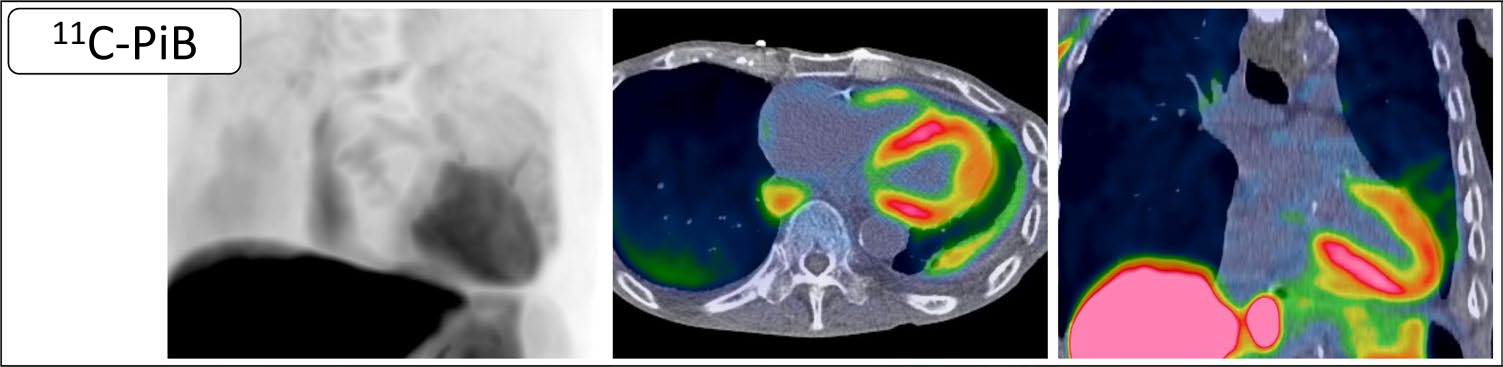
Representative case of AL cardiac amyloidosis evaluated with ^11^C-Pittsburgh compound B (PiB) PET. A male patient in his 70 s was diagnosed with AL cardiac amyloidosis based on biopsy findings. Shown from left to right are maximum intensity projection (MIP), axial PET/CT, and coronal PET/CT images. Diffuse radiotracer uptake is evident in both the left and right ventricular myocardium, with mild uptake also observed in the atrial walls

A major advantage lies in its ability to directly detect amyloid deposits without the need for invasive procedures such as endomyocardial biopsy [60]. Additionally, amyloid PET holds potential for differentiating between various forms of amyloidosis, particularly ATTR-CM and AL-CM (Fig. 4), which require distinct treatments. A study by Rosengren et al. reported higher ^11^C-PiB uptake in AL-CM than in ATTR-CM, suggesting its potential for subtype differentiation despite the small sample size [61]. Although technetium-based scintigraphy is commonly used to detect ATTR-CM, PET imaging could provide enhanced accuracy and earlier detection of amyloid deposits. Furthermore, combining ^11^C-PiB PET with ^99m^Tc-PYP or ^99m^Tc-DPD scintigraphy may be useful for more detailed subtyping of amyloid pathology [62, 63]. Amyloid PET also offers the ability to quantify the myocardial amyloid burden, providing insight into disease severity and progression over time. Such quantification is especially important for monitoring how the disease evolves and responding to changes in the patient's condition [64].

While amyloid PET is considered a promising diagnostic tool for cardiac amyloidosis, a previous report noted limited visualization in some cases [64]. Therefore, optimized imaging protocols including appropriate acquisition timing are needed to enhance diagnostic performance.

### Cardiac magnetic resonance imaging

**Fig. 5:**
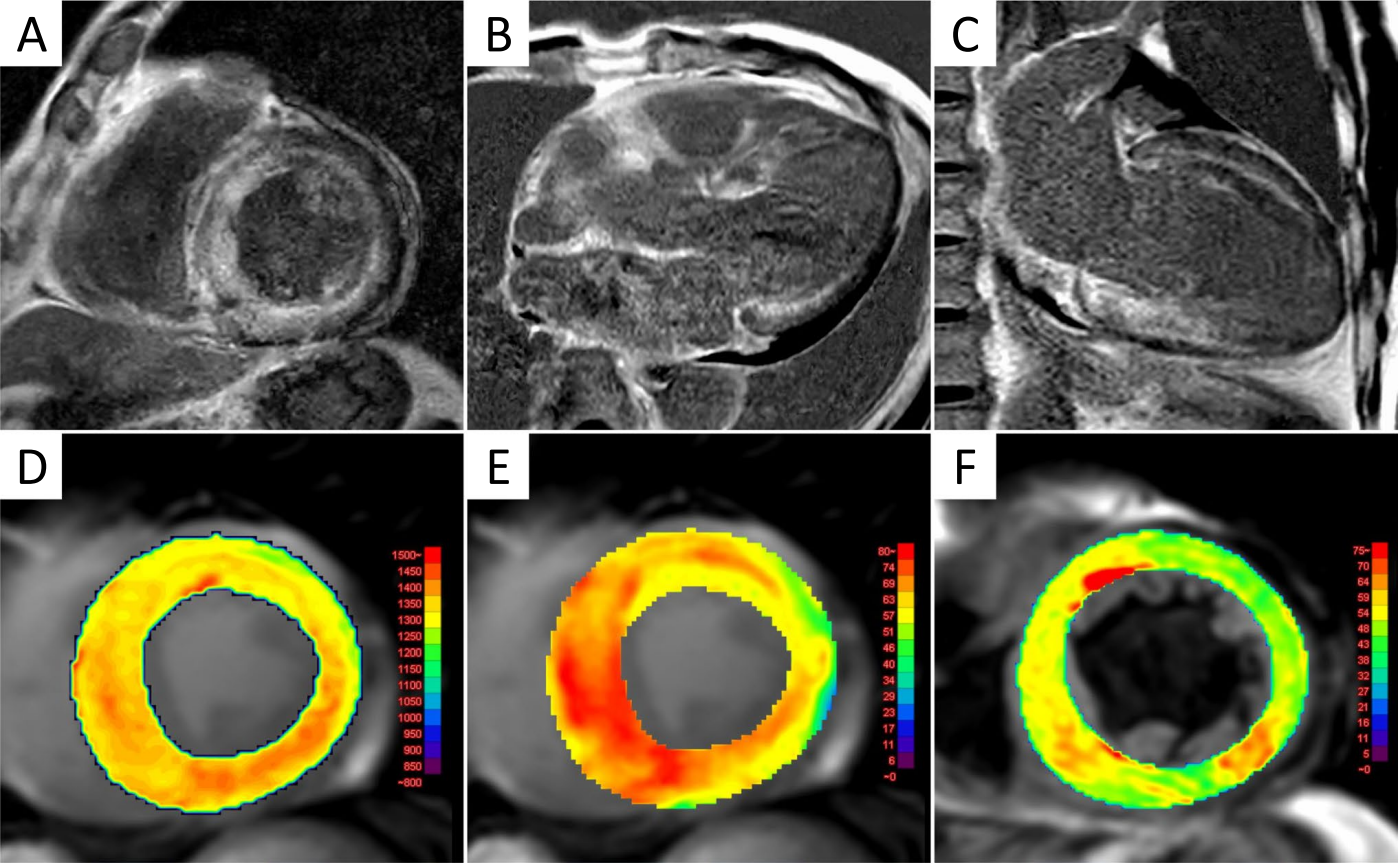
CMR findings in ATTRwt-CM. Cardiac magnetic resonance (CMR) images from a man in his 70 s with wild-type transthyretin cardiac amyloidosis (ATTRwt-CM). Late gadolinium enhancement (LGE) images in the left ventricular short-axis (**A**), 3-chamber (**B**), and 2-chamber (**C**) views show diffuse subendocardial LGE in the left ventricle, with additional LGE in the right ventricular wall and atrial walls. A dark blood pool sign, characterized by low signal intensity within the cardiac chambers, is also observed. Native T1 map (**D**) and extracellular volume (ECV) map (E) demonstrate markedly elevated values (yellow to red areas), consistent with widespread amyloid infiltration. T2 map (F) shows moderately prolonged T2 values

CMR plays a vital role in the diagnosis and management of cardiac amyloidosis by offering comprehensive insights into both myocardial structure and function. One of its major strengths lies in its ability to characterize tissue composition with high precision using late gadolinium enhancement (LGE), T1 and T2 mapping, and extracellular volume (ECV) quantification (Fig. 5). In addition to these advanced tissue characterization capabilities, CMR also serves an important clinical role in ruling out cardiac amyloidosis and differentiating it from other forms of cardiomyopathy.

Recent meta-analyses evaluating the diagnostic performance of LGE, native T1, and ECV for cardiac amyloidosis using CMR have yielded consistent findings [65, 66]. ECV demonstrated the highest diagnostic accuracy, exhibiting superior sensitivity (90%) and specificity (91%) compared with native T1 (sensitivity: 87%, specificity: 88%) and LGE (sensitivity: 85%, specificity: 92%; significantly lower diagnostic odds ratio compared to ECV). On the other hand, native T1 does not require gadolinium-based contrast administration, making it particularly advantageous for assessing myocardial involvement in patients with renal impairment [67].

T2 mapping reflects myocardial water content, and thus elevated T2 values are indicative of myocardial edema or inflammation. Patients with cardiac amyloidosis generally exhibit increased myocardial T2 values, with AL-CM demonstrating higher values compared to ATTR-CM. Nonetheless, patients with ATTR-CM also exhibit elevated T2 values compared with healthy individuals [68, 69]. These findings suggest that T2 mapping may have clinical utility, particularly in the diagnosis of AL-CM [70]. On the other hand, recent studies have suggested that MRI-derived ECV may not accurately reflect the histological amyloid burden in ATTR-CM patients exhibiting prolonged T2 values, a limitation that warrants careful consideration [71, 72].

CMR allows precise evaluation of myocardial deformation through strain analysis using feature-tracking techniques on cine images. In patients with cardiac amyloidosis, global longitudinal strain (GLS) is often markedly impaired, providing incremental prognostic value beyond traditional parameters. Moreover, a pattern of relative apical sparing—characterized by more pronounced strain reduction at the basal and mid-ventricular segments compared to the apex—is frequently observed in cardiac amyloidosis and aids in differentiating it from other hypertrophic cardiac conditions such as hypertrophic cardiomyopathy and Anderson-Fabry disease [73]. This strain pattern commonly correlates with a base-to-apex gradient in LGE and ECV, reflecting regional variations in amyloid deposition [74]. However, the diagnostic accuracy of this relative apical sparing pattern alone is not consistently high and has been reported to be inferior to that of native T1 mapping and ECV [75]. The combination of T2 mapping and LGE has been reported to differentiate AL-CM from ATTR-CM with high accuracy, underscoring the clinical utility of a multiparametric approach [76].

In the field of CMR, AI-driven diagnostic approaches have garnered considerable attention. One reason is that LGE can exhibit diverse and often subtle patterns, making visual interpretation and pattern recognition challenging even for experienced clinicians. A convolutional neural network trained on LGE images demonstrated excellent performance in distinguishing cardiac amyloidosis from other cardiomyopathies, achieving a sensitivity of 95%, diagnostic accuracy of 88%, and an AUC of 0.982—comparable to expert human readers [77]. More recently, a multicenter study applied a Vision Transformer based deep learning model to cine MRI images for the diagnosis of cardiac amyloidosis, achieving AUCs of 0.954 in internal validation and 0.957 in external validation cohorts. Notably, this model proved particularly effective in cases with equivocal imaging findings, highlighting its potential as an objective second-opinion tool and a valuable aid in clinical decision-making [78]. The continued advancement of AI-based approaches holds great promise for enhancing the diagnostic accuracy and efficiency in the evaluation of cardiac amyloidosis [79].

### Cardiac computed tomography

**Fig. 6:**
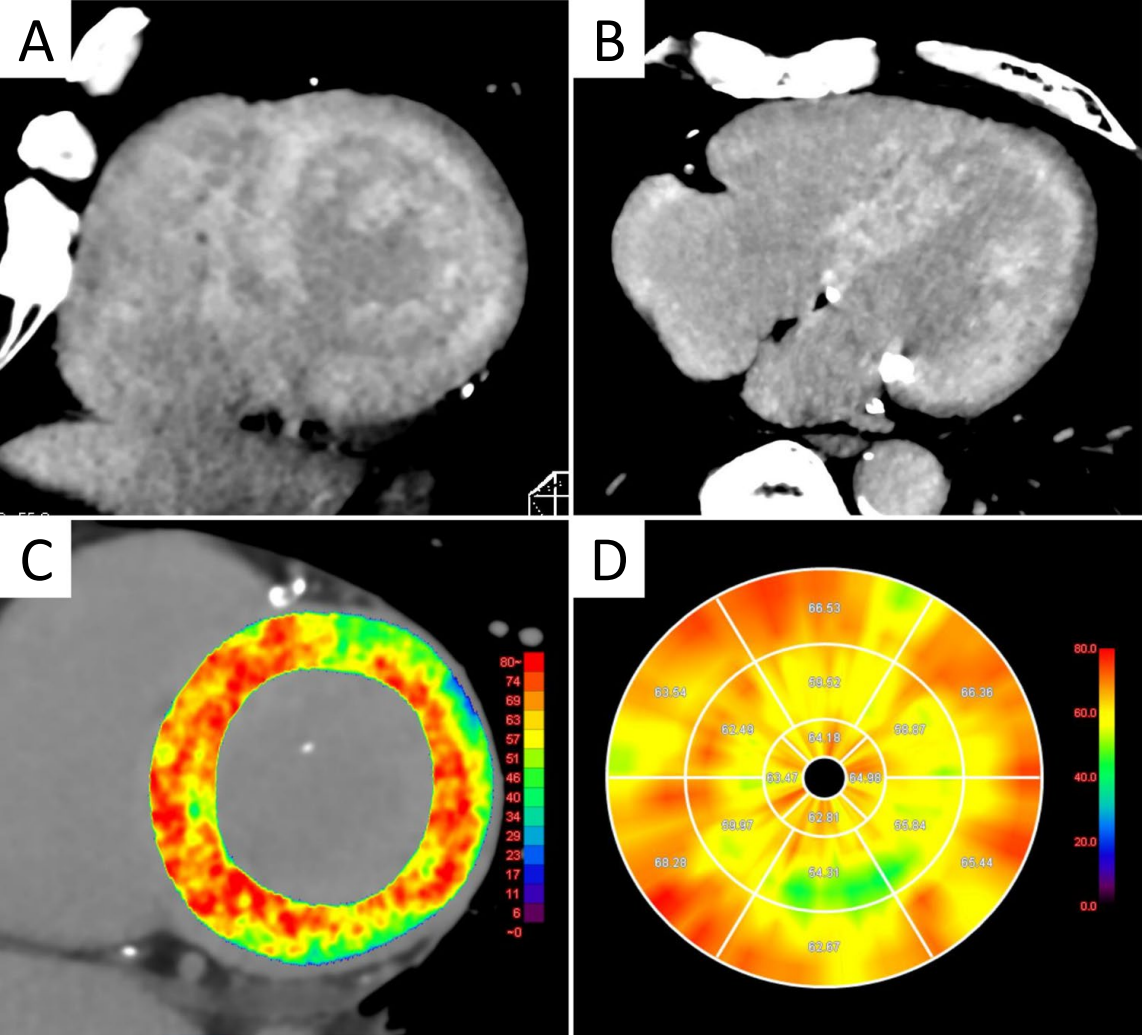
CT findings in ATTRwt-CM. A man in his 70 s with wild-type transthyretin cardiac amyloidosis (ATTRwt-CM). The patient was scheduled for transcatheter aortic valve replacement (TAVR) due to severe aortic stenosis. Preprocedural planning CT with additional late iodine enhancement (LIE) imaging revealed diffuse LIE in the left ventricular (LV) wall (**A**, **B**). CT-derived extracellular volume (ECV) maps demonstrated markedly elevated ECV values (yellow to red areas in **C**, **D**). Subsequent evaluation confirmed concomitant ATTRwt-CM

Although CCT is not indispensable for the diagnosis of cardiac amyloidosis, it serves as a useful alternative for myocardial tissue characterization and functional assessment, particularly when MRI is contraindicated or unavailable. Furthermore, when CCT is performed for other clinical indications, opportunistic evaluation of myocardial tissue may aid in the incidental detection or screening of cardiac amyloidosis. In clinical practice, CCT is mainly used for coronary assessment, while CMR remains the gold standard for myocardial tissue characterization. However, MRI has practical limitations, including long scan times, device-related restrictions, contrast contraindications in dialysis patients, and the need for technical expertise. In contrast, CCT is widely accessible and feasible in many settings. Recent advances now allow CCT to assess myocardial tissue via ECG-gated equilibrium-phase imaging, enabling evaluation of late iodine enhancement and ECV [6, 80, 81]. Notably, CT-derived ECV has proven particularly useful for detecting cardiac amyloidosis and is now recognized as an alternative to CMR in the Japanese guidelines for the diagnosis and management of cardiac amyloidosis [9]. Cardiac amyloidosis is frequently coexistent with conditions such as AS and AF. The addition of ECV assessment to transcatheter aortic valve replacement planning CCT enables effective detection of subclinical cardiac amyloidosis (Fig. 6) [82]. Recent studies have demonstrated that CT-ECV can reliably differentiate between isolated AS, AS with concomitant ATTR-CM, and isolated ATTR-CM with good diagnostic accuracy and defined threshold values [83]. Furthermore, a prospective study showed that incorporating ECV assessment into planning CT for catheter ablation in patients with atrial arrhythmias, in combination with clinical red flags, facilitates early identification of subclinical ATTR-CM [84]. Additionally, emerging evidence supports the utility of photon-counting CT for ECV evaluation in patients with cardiac amyloidosis, highlighting its potential for further advancement and clinical application in the near future [85, 86].

## Diagnostic approach to cardiac amyloidosis

**Fig. 7:**
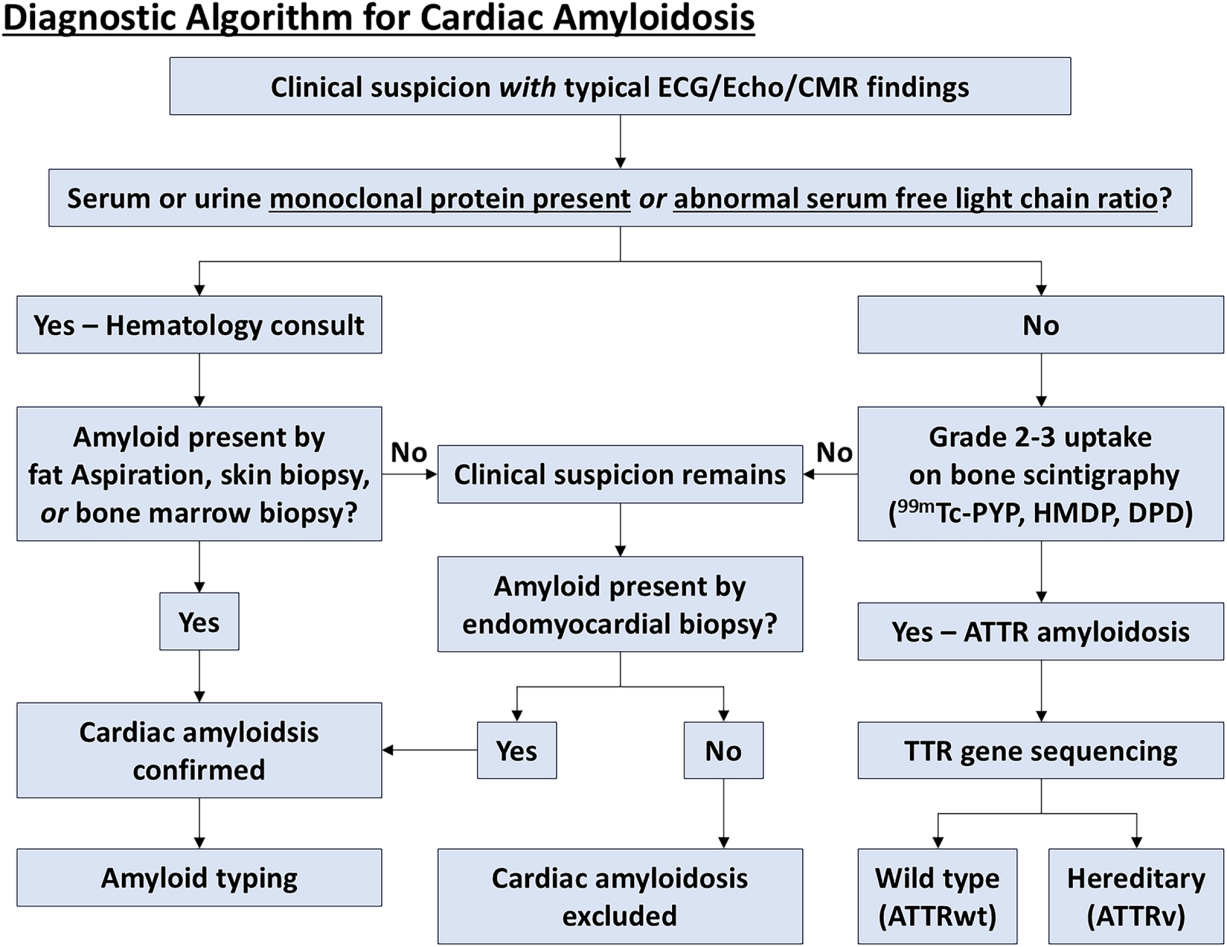
Diagnostic algorithm for cardiac amyloidosis. This flowchart is modified from Hanna et al. [114]. Patients with typical echocardiographic or cardiac magnetic resonance imaging (CMR) findings of cardiac amyloidosis should undergo evaluation for serum and urine protein electrophoresis with immunofixation and serum free light chain assay to assess light chain (AL) amyloidosis. If AL amyloidosis can be ruled out, Grade 2–3 myocardial uptake on bone scintigraphy using ^99m^Tc-pyrophoshate (PYP), ^99m^Tc-hydroxymethylene diphosphonate (HMDP), or ^99m^Tc-3,3-diphosphono-1,2-propanodicarboxylic acid (DPD) can lead to a diagnosis of transthyretin (ATTR) cardiac amyloidosis without a tissue biopsy. SPECT/CT or fusion of SPECT and CT images is recommended to localize myocardial uptake and to exclude blood pool artifacts

An accurate and timely diagnostic approach is essential for cardiac amyloidosis, given the significant differences in prognosis and treatment between AL and ATTR subtypes. This section outlines a stepwise diagnostic algorithm that integrates clinical findings, laboratory testing, and multimodality imaging to guide appropriate subtype identification and management (Fig. 7) (Table 2) [32].

**Table 2:** Imaging differences between ATTR and AL cardiac amyloidosis

Modality	ATTR amyloidosis	AL amyloidosis
^99m^Tc-labeled phosphate scintigraphy	Strong myocardial uptake (Perugini grade 2–3); high H/CL ratio (≥ 1.5 at 1 h); low false-positive rate when monoclonal protein is excluded	Minimal or absent uptake; Perugini grade 0–1; false positives rare but possible, especially with myocardial injury or concurrent pathologies
PET (^18^F-florbetapir, ^18^F-florbetaben, ^18^F-flutemetamol, ^11^C-PiB)	Mild to moderate myocardial uptake; variable depending on genotype (ATTRwt vs. ATTRv)	Strong and diffuse myocardial uptake; high sensitivity and specificity; superior to scintigraphy in some AL cases
CMR	Subendocardial or diffuse LGE; very high ECV (> 45–50%); mildly elevated native T1 and T2 values	Global or transmural LGE; elevated native T1 and markedly prolonged T2 (inflammation/edema); increased ECV; less frequent apical sparing pattern
CCT	LIE; increased CT-derived ECV; applicable in TAVR/ablation planning	May show LIE and increased ECV; less validated and less commonly used in AL amyloidosis evaluation

ATTR, transthyretin amyloidosis; AL, light chain amyloidosis; PET, positron emission tomography; CMR, cardiac magnetic resonance; CCT, cardiac computed tomography; H/CL, heart-to-contralateral lung ratio; LGE, late gadolinium enhancement; ECV, extracellular volume; T1/T2, MRI relaxation times; PiB, Pittsburgh Compound B; LIE, late iodine enhancement; CAD, coronary artery disease; PYP, pyrophosphate; ATTRwt, wild-type ATTR; ATTRv, variant (hereditary) ATTR

The diagnostic process begins with clinical suspicion, which should be raised in patients with unexplained LV hypertrophy, particularly in the absence of hypertension or valvular disease. Additional red flags include heart failure with preserved LVEF, low-QRS voltage on ECG despite thickened ventricular walls on echocardiography, and extracardiac manifestations such as bilateral carpal tunnel syndrome, lumbar spinal stenosis, and spontaneous biceps tendon rupture. AF with concentric LV hypertrophy or elderly patients undergoing evaluation for AS should also prompt consideration of cardiac amyloidosis, as these populations have an elevated prevalence of ATTR-CM [87].

Upon clinical suspicion, the next step is to confirm the presence of amyloid deposition. This is typically achieved by Congo red staining of tissue biopsies viewed under polarized light microscopy, which reveals characteristic apple-green birefringence. While endomyocardial biopsy remains the gold standard, less invasive options such as abdominal fat pad aspiration, salivary gland, or rectal mucosa biopsy can often be used. However, in many cases of ATTR-CM, especially wild-type forms, a non-biopsy diagnostic pathway can be pursued. Regardless of biopsy findings, amyloid typing is essential. In all patients with suspected cardiac amyloidosis, laboratory testing should include serum and urine protein electrophoresis with immunofixation and a serum FLC assay to detect the presence of monoclonal protein. A positive result indicates AL amyloidosis and necessitates urgent hematologic evaluation and treatment due to its aggressive nature [41].

If AL amyloidosis is excluded by the absence of monoclonal protein, the diagnostic algorithm shifts toward confirmation of ATTR-CM. This involves bone scintigraphy using technetium-labeled tracers such as ^99m^Tc- PYP, ^99m^Tc-DPD, or ^99m^Tc-HMDP, depending on regional availability. Interpretation of the scan relies on the Perugini grading system, which compares cardiac uptake to that of adjacent bone. Grades 0 and 1 are considered negative or indeterminate, while Grade 2 (equal to bone) and Grade 3 (greater than bone with reduced bone signal) are strongly suggestive of ATTR-CM. In the appropriate clinical context, a diagnosis of ATTR-CM can be made without tissue biopsy. This approach is endorsed by current international guidelines and is reflected in the 2020 Japanese Circulation Society (JCS) guidelines, which permit a non-biopsy diagnosis of wild-type ATTR-CM (ATTRwt-CM) when all of the following criteria are fulfilled: [1] Grade 2 or 3 myocardial uptake on bone scintigraphy; [2] no evidence of monoclonal protein on serum/urine immunofixation and serum FLC assay; and [3] absence of pathogenic TTR gene mutations. Importantly, alternative causes of radiotracer accumulation—such as myocardial infarction, cardiac sarcoidosis, or valvular/annular calcification—must also be excluded [9, 41, 88].

In cases where findings are equivocal (e.g., Grade 1 uptake), or when both monoclonal protein and radiotracer uptake are present (possible dual pathology), further assessment with　CMR or endomyocardial biopsy may be necessary. CMR provides valuable information on myocardial tissue characteristics, including LGE, native T1 values, and ECV, all of which support the diagnosis and stratification of amyloid burden [71].

## Monitoring treatment response in cardiac amyloidosis

Imaging plays a central role in assessing treatment response in cardiac amyloidosis, offering a non-invasive means to monitor disease activity, track therapeutic efficacy, and inform prognosis. Recent advances in echocardiography, nuclear scintigraphy, and CMR have enhanced the precision and reproducibility of such assessments (Table 3).

**Table 3:** Treatment response by imaging modality

Modality	Key parameters	Response indicators
Echocardiography	GLS, RV free wall strain, diastolic function	Improved GLS or RV strain, improved diastolic filling
SPECT/CT	SUV, % Injected Dose, SUV volume	↓SUV, ↓SUV volume, ↓% Injected Dose (especially with tafamidis or RNAi therapy)
CMR	ECV, T1/T2 mapping, LGE, strain (GLS)	↓ECV, ↓T2 (if edema resolves), improved GLS
CCT	CT-derived ECV, delayed iodine enhancement	↓ ECV or stable values over time

GLS, global longitudinal strain; RV, right ventricle; SPECT/CT, single-photon emission computed tomography/computed tomography; SUV, standardized uptake value; RNAi, ribonucleic acid interference; CMR, cardiovascular magnetic resonance; CCT, cardiac computed tomography; ECV, extracellular volume; T1/T2, MRI relaxation times; LGE, late gadolinium enhancement; CT, computed tomography

While echocardiography is widely used in clinical practice, its role in treatment response monitoring is evolving. Speckle-tracking echocardiography allows for the assessment of GLS and RV free wall strain, both of which are sensitive to functional changes in cardiac amyloidosis. Improvements in RV strain have been associated with cardiac response in AL-CM, and GLS may detect subclinical dysfunction earlier than LVEF in ATTR-CM [89]. While further validation is needed, echocardiographic strain metrics are increasingly integrated into multiparametric follow-up protocols.

Quantitative SPECT/CT using bone-avid tracers such as ^99m^Tc-PYP and ^99m^Tc-DPD allows for objective monitoring of amyloid burden in ATTR-CM. In a prospective study, patients treated with tafamidis demonstrated significant reductions in total SUV, SUV volume, and percent injected dose over a 6- to 12-month period—equating to an approximate 7.7% monthly decline in amyloid tracer uptake [51]. Similar results were observed in patients receiving RNA interference therapies (e.g., patisiran, inotersen), with significant reductions in myocardial SUV retention indices [90]. These SUV metrics correlate well with clinical severity, biomarkers, and CT-derived ECV [54, 91]. Moreover, higher tracer uptake has been linked to worse outcomes, such as reduced LVEF and increased heart failure hospitalizations [55]. A recent observational study following tafamidis-treated ATTR-CM patients for 6 months revealed that progression was most frequently detected in the imaging domain, particularly via worsening diastolic function and strain values—often preceding clinical symptoms or biomarker elevation [92].

Amyloid PET also plays a role in monitoring the response to treatment. As treatments aim to reduce or stabilize amyloid deposits, PET imaging can provide a visual and quantitative assessment of how amyloid accumulation changes during therapy. This allows clinicians to gauge the effectiveness of the treatment and make informed decisions about ongoing care strategies [58, 93].

In patients with AL-CM, multiparametric CMR has become a cornerstone for evaluating therapeutic response. Among the most promising imaging biomarkers, ECV mapping and T2 relaxation time have gained attention for their ability to reflect changes in myocardial amyloid burden and tissue characteristics. In a prospective study of 111 patients, reductions in both LV and RV ECV were observed following chemotherapy among those who achieved favorable hematologic and cardiac biomarker responses. Improvements in RV strain and slight increases in T2 values were also reported, suggesting potential reversal of myocardial edema and functional recovery [94]. Another prospective study confirmed that reductions in LV and RV ECV correlated with hematologic response and improvements in biomarkers, T2 values, and strain parameters, further supporting the role of ECV as an imaging marker of amyloid burden regression and myocardial recovery in AL-CM [95]. In ATTRv-CM treated with patisiran, serial CMR assessments showed significant reductions in ECV over 12 months, validating its utility as a surrogate marker for amyloid regression [96]. Similarly, a recent study demonstrated that ECV remained stable or showed mild improvement over a 12-month period in patients with ATTR-CM treated with tafamidis, indicating disease stabilization [97, 98]. Furthermore, in the first-in-human phase 1 trial of NI006—a monoclonal antibody targeting amyloid fibrils—dose-dependent reductions in ECV were observed after 3 to 6 months of therapy, underscoring the value of ECV as a sensitive early indicator of anti-amyloid treatment efficacy [99].

## Prognostic imaging markers in cardiac amyloidosis

Non-invasive imaging plays a pivotal role in the prognostic assessment of cardiac amyloidosis, providing insights into myocardial infiltration, functional impairment, and overall disease burden. Recent advances across echocardiography, nuclear imaging, CMR, and CCT have identified several imaging biomarkers that independently predict adverse outcomes and assist in risk stratification (Table 4).

**Table 4:** Prognostic markers in cardiac amyloidosis imaging

Modality	Prognostic markers
Echocardiography	Apical sparing pattern: suggests better preserved function Reduced RV free wall strain: predicts worse outcomes Myocardial work indices and GLS dispersion: associated with adverse prognosis
SPECT/CT	High myocardial SUVmax or retention index: associated with worse outcomes RV uptake: linked to greater disease burden and mortality Correlates with ECV and LV mass on CMR
CMR	Elevated ECV: associated with reduced survival Prolonged T2 relaxation time: independent predictor of mortality High native T1 values: associated with poor outcome Reduced GLS and LA strain: predictive of MACE

ATTR, transthyretin amyloidosis; AL, light-chain amyloidosis; SPECT/CT, single-photon emission computed tomography/computed tomography; CMR, cardiovascular magnetic resonance; ECV, extracellular volume; T1/T2, MRI relaxation times; SUV, standardized uptake value; SUVmax, maximum standardized uptake value; GLS, global longitudinal strain; LA, left atrial; MACE, major adverse cardiovascular events; LV, left ventricle; RV, right ventricle

Prognostic assessment using speckle-tracking echocardiography has shown that relative apical sparing of longitudinal strain, a characteristic feature of cardiac amyloidosis, is associated with longer survival [100]. Patients with more pronounced apical-to-basal strain gradients exhibit better preserved myocardial function and improved outcomes. Moreover, RV free wall strain and myocardial work indices, including the apical-to-basal work ratio and longitudinal strain dispersion, provide incremental prognostic value beyond conventional parameters such as ejection fraction and wall thickness [101–103].

While initially developed for diagnosis, bone-avid tracer imaging using ^99m^Tc-PYP or ^99m^Tc-DPD has been increasingly applied in prognostic contexts. Quantitative metrics such as SUVmax, myocardial retention indices, and total cardiac SUV load have been associated with clinical status, functional capacity, and mortality risk [54]. Importantly, elevated RV uptake or diffuse myocardial distribution have been linked to more advanced disease and worse outcomes [104]. SUV metrics obtained from SPECT/CT correlate well with myocardial ECV on CMR and LV mass, suggesting their broader value beyond visual Perugini grading [91]. These findings support the integration of quantitative SPECT/CT into risk models, especially in settings where CMR is contraindicated.

CMR-derived myocardial ECV has emerged as one of the most robust predictors of prognosis [105]. In AL-CM, studies have shown that elevated ECV values, particularly above 47–50%, are strongly associated with reduced survival, irrespective of hematologic response [106, 107]. Complementary large-scale data have established baseline ECV as an independent predictor of mortality: patients with ECV > 40% had poor outcomes unless a deep hematologic response was achieved, while those with ECV < 30% had favorable prognosis regardless of treatment response [107]. Furthermore, T2 prolongation (> 44 ms) has been shown to be independently associated with increased mortality, even after adjusting for LGE, LVEF, and hematologic response in AL-CM. This finding suggests that persistent myocardial inflammation or edema may indicate subclinical disease activity despite biochemical remission [106]. Recent studies have also emphasized the prognostic value of combining strain, ECV, and perfusion imaging. Quantitative stress perfusion MRI detects microvascular dysfunction in AL-CM and improves survival prediction when combined with ECV [108]. GLS and ECV have also shown strong associations with major adverse cardiac events, outperforming traditional staging systems such as Mayo stage when incorporated into multiparametric models [109].

In ATTR-CM, CMR also provides robust prognostic markers. A landmark study demonstrated that ECV values exceeding 59% were strongly predictive of adverse outcomes, with native T1 mapping also associated with disease severity and prognosis [110]. In contrast to AL-CM, T2 values in ATTR-CM have not been shown to predict mortality, underscoring distinct pathophysiologic mechanisms between subtypes. Moreover, a recent meta-analysis synthesizing data from CMR studies confirmed that ECV, native T1, and LGE are independently associated with survival, with ECV showing the highest pooled hazard ratio for mortality prediction [111].

Recent advances in CCT have enabled the quantification of CT-derived ECV, which also shows prognostic utility. In two prospective studies, higher CT-ECV was independently associated with all-cause mortality and adverse cardiac events in patients with suspected or confirmed cardiac amyloidosis [112, 113]. These findings support the role of CT as a practical alternative when CMR is contraindicated.

## Conclusion

Cardiac amyloidosis represents a diagnostic and therapeutic challenge due to its heterogeneous presentation and variable prognosis. Advances in multimodality imaging have transformed its evaluation by enabling early detection, non-invasive subtype differentiation, and objective monitoring of treatment response. Integrating these imaging tools into routine clinical practice and clinical trials will be essential for advancing personalized care and improving outcomes in patients with cardiac amyloidosis.

## Data Availability

The datasets used and/or analyzed in the current study are available from the corresponding author upon request.
